# Observations upon the Treatment of Some Cases of Neurasthenia*Read before the St. Louis Medical Society Saturday Evening, February 5, 1898.

**Published:** 1898-06

**Authors:** Jerome K. Bauduy

**Affiliations:** St. Louis, Mo.; Professor Nervous and Mental Diseases, and of Medical Jurisprudence, Missouri Medical College


### OBSERVATIONS UPON THE TREATMENT OF SOME CASES OF NEURASTHENIA.*

BY JEROME K. BAUDUY, M.D., LL.D., ST. LOUIS, MO.

Professor Nervous and Mental Diseases, and of Medical Jurisprudence, Missouri Medical College.

That chalybeates, more especially the *organic* salts of iron, constitute an essential indication in the successful treatment of some cases of neurasthenia, especially in the female where functional menstrual derangements exist, is to my mind *an indisputable fact.* They produce conditions oftentimes not attainable by the inorganic preparations, for many reasons which experience and reflection clearly demonstrate.

In a recent clinical study of this affection my conclusion as above stated is fully justified and corroborated by the microscopical blood examinations conducted by my esteemed and skillful friend, Dr. C. Fisch. That cerebro-spinal anæmia is a frequent important concomitant, if not an essential etiological factor of neurasthenia, I hardly think admits of cavil.

The clinical histories of appended cases were compiled by my son, Dr. Keating Bauduy, chief of the Neurological Clinic at St. John's Hospital, under whose direct supervision the investigations were conducted. That the ratio or number of red blood corpuscles and the percentage of hemoglobin were deficient in the normal standard of these cases prior to the treatment *is incontestable*, as shown by the microscope. That several of the cases to be enumerated showed marked improvement even after one or two weeks' treatment is moreover revealed in the same manner, and which for rapidity of effect is quite an exceptional, if not a startling, therapeutic result when compared with some of the prior and more established methods of treatment. That many of these cases presented unmistakable evidence of satisfactory improvement, from both a subjective and objective standpoint, was quite as notable as the permanent character of their general amelioration. That the ordinary tonics had in some instances been administered with nugatory results while pursued along the old lines of authoritative medication seems quite manifest.

My only explanation of the *surprising results* in the cases herein cited, where the usual officinal class of remedies had formerly been ineffectually essayed, was the superinduction, as is so frequently the case, of disturbed digestion and assimilation; results but too familiar and disappointing to professional experience. Aside from the disturbances just mentioned, the development of headache, constipation, etc., frequently obviate their further administration.

When, a few years ago, my attention was called to Gude's preparation of "*Liquor Mangano-Ferri Peptonatus, Gude*" (Pepto-Mangan), so extensively used and highly extolled in Germany,

---

*Read before the St. Louis Medical Society Saturday Evening, February 5, 1898.

with my usual antipathy for new remedies, I reluctantly gave it a trial, anticipating that I would necessarily have to combat the usual disappointing effects of most of the other preparations of iron. The results, however, were *indeed a surprise to myself*, for the concomitant deranging sequelæ were so slight, that but in very few instances in *my extensive* utilization and experience with this special pharmaceutical preparation was I obliged to discontinue it. My experience having led me to believe that iron and manganese in combination are both indicated in the vast majority of cases of neurasthenia, this particular remedy, *I am now convinced, will prove a great boon both to the patient and the physician.* While it is maintained by some that in the hemoglobin of the red blood corpuscle manganese is present as well as iron, I have for many years procured results with a combination of both not directly obtainable with one alone. We know, however, that manganese gives off oxygen to a greater degree than iron, and it has been argued that for this reason its internal exhibition might correspondingly increase assimilation.

Dr. Fisch's appended microscopical report shows that the increase in the percentage of hemoglobin in many of this series of cases is far in excess of the proportionate increase of the red blood corpuscles. *This fact I deem of greater importance as to the effectiveness of the medicine*, because the count of the blood corpuscles is to a certain extent relative, and the size varies greatly in different cases, and for other reasons the same amount of blood plasma contains different numbers of red cells; hence I would particularly lay stress upon the proportionate increase of the hemoglobin as the more important factor. The *notable* and *astonishing improvement* of these cases when placed upon this preparation led me to their closer scrutiny, as well as microscopic observation. Before concluding, I *wish particularly* to call attention to the fact of the absence of digestive disturbances and necessary consequent interference in the assimilation. All other unpleasant complicating results were notable by their absence. Of course we do not consider the remedy applicable to cases of lithemic neurasthenia, nor in any manner a *specific* in any variety of neurasthenia. In many cases the addition of arsenic and strychnia greatly increases the efficacy of the preparation. I must also take cognizance of the salient fact of the rapidity with which a large number of female neurasthenics under our treatment who have suffered with marked functional menstrual derangements have attained a normal condition under the administration *of this most elegant combination of iron and manganese.*

As it would be tedious and monotonous to present an exhausted citation of multiplicity of a clinical cases, I have confined myself to a recital of a few typical ones:

